# Childhood abuse and neglect in routine care psychotherapy patients

**DOI:** 10.3389/fpsyt.2025.1566560

**Published:** 2025-04-17

**Authors:** Tobias Teismann, Kurt Hahlweg, Sören Friedrich, Jürgen Margraf

**Affiliations:** ^1^ Mental Health Research and Treatment Center, Faculty of Psychology, Ruhr-Universität Bochum, Bochum, Germany; ^2^ DZPG (German Center for Mental Health) Partner Site Bochum/Marburg, Bochum, Germany; ^3^ Department of Clinical Psychology, Technische Universität Braunschweig, Braunschweig, Germany; ^4^ Department of Adolescent Psychology and Psychotherapy, Albert-Ludwigs-Universität Freiburg, Freiburg, Germany

**Keywords:** childhood maltreatment, emotional abuse, emotional neglect, psychotherapy, cognitive behavior therapy

## Abstract

**Background:**

Childhood maltreatment has been well established to contribute to the development and the poorer course of mental disorders across the lifespan. However, studies focusing on patients who are undergoing psychotherapy in natural settings are rare. On this background, the current study aimed to investigate (1) the prevalence of childhood maltreatment in routine care psychotherapy patients, (2) associations between childhood maltreatment and symptom severity, and (3) the influence of childhood maltreatment on treatment outcome.

**Method:**

Data from *N* = 549 outpatients [60.3% female; age: *M(SD)* = 36.29 (13.47), range: 17–74 years] who received cognitive behavioral therapy at an outpatient clinic were collected. Self-report measures of childhood maltreatment, depression, anxiety, positive mental health, and treatment satisfaction were assessed before and after treatment.

**Results:**

Any form of childhood maltreatment was reported by 57.6% of the study sample; women were more affected than men, and childhood maltreatment was associated with heightened symptom severity and lowered positive mental health. Emotional abuse was predictive of increased posttreatment depression, anxiety, and reduced positive mental health, whereas emotional neglect was predictive of lower patient-reported global treatment success—after controlling for age, gender, pretreatment depression, anxiety, and positive mental health.

**Discussion:**

Childhood maltreatment is prevalent in routine care psychotherapy patients and associated with symptom severity as well as reduced treatment response. Emotional abuse and emotional neglect exert an especially pernicious influence; particular attention must therefore be paid to these respective childhood experiences, as they can easily go unnoticed in the early phases of psychotherapy.

## Background

Childhood maltreatment has been well established to contribute to the development and the poorer course of mental disorders across the lifespan (1). Childhood maltreatment is commonly defined as “all forms of physical and/or emotional ill-treatment, sexual abuse, neglect or negligent treatment or commercial or other exploitation, resulting in actual or potential harm to the child’s health, survival, development or dignity in the context of a relationship of responsibility, trust or power” (2). It is conceptualized as emotional, physical, and sexual abuse, or emotional and physical neglect before the age of 18 (3). It is estimated that one in four children will experience child abuse or neglect at some point in their lifetime and one out of seven children have experienced abuse over the last year. However, it is widely accepted that these statistics represent a significant underestimate of the prevalence of childhood maltreatment because the majority of abuse and neglect goes unreported (4).

Childhood maltreatment has severe and long-lasting effects on both mental and somatic health across the lifespan. About one-third of all adult-onset psychiatric disorders are related to childhood maltreatment, with effects on morbidity and mortality. It is associated with an earlier age of onset and more severe clinical course (i.e., symptom severity) and poorer treatment response to pharmaco-/psychotherapy (5, 6). Childhood maltreatment not only affects mental health but also increases the risk of obesity, diabetes, lung disease, and cardiovascular disorders (4, 7). Unfortunately, childhood maltreatment is common in the general population. For instance, a recent systematic review on the lifetime prevalence of self-reported childhood maltreatment revealed the following median prevalence rates for European countries: Sexual abuse 13.2%, emotional abuse 11.7%, physical abuse 12.2%, and neglect 27.0% (8).

In clinical samples, the prevalence rates are higher as evidenced in two meta-analyses: Nelson et al. (6) estimated the prevalence of different types of childhood maltreatment in adults suffering from depression. Kuzminskaite et al. (5) used data from the “Netherlands Study of Depression and Anxiety NESDA” including patients suffering from current and remitted depressive and/or anxiety disorders to calculate prevalence rates. In the Nelson et al. study, 45.6% of the sample had experienced at least one type of childhood maltreatment; in the Kuzminskaite et al. study, it was 53.9%; with emotional neglect (43.2%/44.0%) and emotional abuse (36.7%/28.5%) being the most prevalent types, followed by physical abuse (27.6%/15.4%), and sexual abuse (25.3%/19.7%).

Nelson et al. (6) also reported that all types of childhood maltreatment significantly increased the risk of adult depression in individuals with childhood maltreatment compared with those without childhood maltreatment. Furthermore, depressive disorder is twice as likely to take a chronic course in individuals with a history of childhood maltreatment, and childhood maltreatment has been shown to be associated with symptom severity (3, 5, 6, 9). Finally, evidence-based pharmacotherapies and psychotherapies seem to be less effective in patients with a history of childhood maltreatment compared to patients without childhood maltreatment, especially for depressed patients: In a meta-analysis by Nanni et al. (10), adults and adolescents with depression and childhood maltreatment were about 1.5 times more likely not to respond or remit after pharmacotherapy, psychotherapy, or combination treatment than depressed patients with no such experiences. These findings were extended by a meta-analysis of studies published in 2013, which suggested that adults with depression and childhood maltreatment were twice as likely not to respond to psychotherapy or pharmacotherapy (6). However, treatment outcomes in patients with childhood maltreatment have not been definitive. In the most recent meta-study by the Childhood Trauma Meta-Analysis Study Group (3), 29 trials—both RCTs and open trials—with depressed patients with and without childhood maltreatment were included. More than half (62%) of the patients reported a childhood maltreatment history. Treatment was efficacious in reducing depression severity for both individuals with and those without childhood maltreatment (*vs.* control condition). As such, despite having more severe depression at baseline, there was no significant difference in active treatment effects between individuals with and without childhood maltreatment. Inconsistent findings within these meta-analyses might be due to publication and selection bias (3) as well as different operationalization of therapy outcome (9).

To summarize, childhood maltreatment is a common risk factor for developing in particular depressive and anxiety disorder in adulthood. The prevalence of childhood maltreatment in patient samples is about 50%. All types of childhood maltreatment, predominantly emotional abuse/neglect, seem to be associated with psychological distress. Furthermore, childhood maltreatment is associated with earlier onset, more chronic or recurrent symptoms, and greater probability of having comorbidities, especially in depressed patients. However, very different samples have been included in the various trials, and up to date, there are only few studies focusing on patients who are undergoing psychotherapy in natural settings (9), which are not part of research projects with the usual restrictions (exclusion and inclusion criteria to sample homogenous patients, following strict treatment manuals, same length of treatment). Here we will focus on such a sample from an outpatient clinic with routine outcome monitoring. The following questions will be answered: Are there gender differences in terms of childhood maltreatment prevalence rates? Do different diagnostic groups differ in terms of childhood maltreatment type prevalence rates? Is symptom severity associated with childhood maltreatment? Are there differences in treatment outcome depending on the presence of childhood maltreatment? Consistent with previous studies, we expected (1) women to report more experiences with childhood maltreatment than men and (2) childhood maltreatment to be associated with symptom severity. No expectations were formulated with regard to the diagnostic groups, mainly because it was initially unclear which diagnostic groups would be available in sufficient numbers in the current sample. Furthermore, due to inconsistent findings (3, 6, 9, 10), no specific expectations were formulated with regard to maltreatment-related differences in treatment outcome.

## Materials and methods

### Participants

The study sample comprises *N* = 549 adult outpatients [60.3% female; age: *M(SD)* = 36.29 (13.47), range: 17–74 years] undergoing individual cognitive behavioral therapy at an outpatient university clinic in the Ruhr area in Germany between April 2017 and September 2022. Participants were eligible for the current study if complete CTQ data were available. Assessments were conducted prior to treatment (T1) and again after 24 therapy sessions [T2; mean time between T1 and T2: (*M*) = 9.27 months, standard deviation (*SD*) = 3.22; number of therapy sessions: (*M*) = 23.60 sessions, (*SD*) = 1.43], which corresponds to a short-term therapy within the German insurance system. The most common primary diagnoses according to the International Classification of Diseases (ICD-10) were Recurrent Depressive Disorder (21.9%, *n* = 120), Adjustment Disorder (10.9%, *n* = 60), Single Episode Depressive Disorder (10.0%, *n* = 55), Social Phobia (9.5%, *n* = 52), Panic Disorder with/without Agoraphobia (9.1%, *n* = 50) and Dysthymia (7.8%, *n* = 43). Comparable few participants suffered from Posttraumatic Stress Disorder (PTSD; 4.9%, *n* = 27) or Borderline Personality Disorder (BPS; 3.1%, *n* = 17). All patients were Caucasian. Treatments were conducted by clinical psychologists in advanced CBT training, who provided treatment under close-meshed CBT supervision (every fourth session with a licensed CBT supervisor). Treatments generally followed published CBT guidelines for each disorder but were, in most cases, less standardized than in RCTs. Treatments were paid for by the German health insurance system.

All patients were informed that the clinic regularly conducts research and provided informed consent prior to participation. In order to assure a standard of quality, all clients seeking help at the clinic are required to fill out questionnaires prior to their intake. No compensation is given to clients for doing so. This study was reviewed and approved by the local ethics committee (318/2016).

### Measures

Childhood Trauma Questionnaire (CTQ; 11). The CTQ is a retrospective self-report measure of adverse childhood experiences. The scale includes 28 items assessing sexual (SA, “made to do sexual things”), emotional (EA, “felt hated by family”) and physical abuse (PA, “hit hard enough to see a doctor”), and emotional (EN, “felt loved”) and physical neglect (PN, “wore dirty cloth”) with five items per subscale (and three items assessing dissimulation tendencies). All items are to be answered on a 5-point Likert scale ranging from “1” (never) to “5” (very often). The subscales of the German version of the CTQ (11) have been shown to have high internal consistency (α ≥.80) except for the subscale physical neglect (*α* = .55). Accordingly, internal consistency was good in the current sample: EA (*α* = .88), PA (*α* = .86), SA (*α* = .91), EN (*α* = .91), and PN (*α* = .64). Maltreatment is assumed when threshold scores for emotional abuse (≥10), emotional neglect (≥15), physical abuse (≥8), physical neglect (≥8), and sexual abuse (≥8) are met (12). Furthermore, the CTQ manual (13) contains thresholds to divide subscale scores into severity quartiles: “none/minimal” (EA < = 8, PA < = 7, SA = 5, EN < = 9, PN< = 7), “low to moderate” (EA > 8 and < = 12, PA > 7 and < = 9, SA > 5 and < = 7, EN > 9 and < = 14, PN > 7 and < = 9), “moderate to severe” (EA > 12 and < = 15, PA > 9 and < = 12, SA > 7 and < = 12, EN > 15 and < = 17, PN > 9 and < = 12), and “severe to extreme” (EA > = 16, PA > = 13, SA > = 13, EN > = 18, PN > = 13). Due to a technical error, one item of the sexual abuse subscale (“someone molested me”) was not presented to participants. Therefore, the total score of this subscale cannot be compared to other studies, and prevalence scores on sexual abuse cannot be reported in the current study.

Depression Anxiety Stress Scales (DASS-42 (14);). The DASS measures symptoms of depression, anxiety, and stress over the past week with 14 items per subscale. In the current study, only the depression subscale (e.g., “I felt that life was meaningless”) and the anxiety subscale (e.g., “I felt scared without any good reason”) were used. All items are rated on a 4-point Likert-type scale (0 = *did not apply to me at all*, 3 = *applies to me very much or most of the time*). Higher sum scores indicate more severe symptoms. All subscales have been shown to have high internal consistency (DASS-D: *α* = .81–.88; DASS-A: *α* = .78–.84 (15);). Accordingly, internal consistency was good in the current sample: DASS-D: *α*= .95 (T1) and *α*= .96 (T2); DASS-A: *α*= .88 (T1) and *α*= .91 (T2).

Positive Mental Health Scale (16). The PMH scale asks respondents to rate how much they agree with different statements on subjective and psychological well-being (e.g., “I enjoy my life”, “All in all, I am satisfied with my life”, “I feel that I am actually well equipped to deal with life and its difficulties”). The scale comprises nine items rated on a scale ranging from 1 (*do not agree*) to 4 (*agree*), with higher scores indicating greater positive mental health. Unidimensional structure, good convergent, and discriminant validity as well as high internal consistency (*α* >.81) have been demonstrated in various populations (16). Accordingly, Cronbach’s alpha was good in the current study: *α*=.90. (T1) and *α*= .93 (T2).

Global success ratings (17). Patients’ evaluation of their global therapy success was assessed with two items (“Have your expectations of the therapy been fulfilled”, “How much did the therapy benefit you overall?”) that are rated on a scale ranging from 1 (“on the opposite—it has become worse”) to 6 (“completely/very much”). Higher scores represent higher satisfaction with treatment. The two items are strongly correlated (*r* = .737, *p* <.001) and were summed up in the current study (18).

### Statistical analyses

Statistical analyses were conducted using SPSS 29. Maltreatment prevalence rates will be reported according to the cut-off scores established by Walker et al. (12): CTQ emotional abuse (≥10), CTQ emotional neglect (≥15), CTQ physical abuse (≥8), and CTQ physical neglect (≥8). Furthermore, maltreatment rates will be reported in accordance with the severity quartiles established by Bernstein et al. (13). In addition, rates of maltreatment are going to be reported separately for male and female gender, as well as for the different diagnostic groups. Assignment to a diagnostic group (yes/no) was based on the respective primary diagnosis. Only mental disorders with a prevalence rate 5% within the current sample were taken into account. Gender differences in the severity of maltreatment were investigated using *t*-test for independent samples. A series of multiple logistic regression analysis were used to examine the relative contribution of diagnostic groups, age, and gender to the prediction of the presence of emotional abuse, physical abuse, emotional neglect, and physical neglect. Associations between pretreatment symptom severity (DASS-D, DASS-A, PMH) and childhood maltreatment were established using correlation analyses. Changes in study variables from T1 to T2 were analyzed using *t*-tests for dependent samples. To identify significant predictors of posttreatment (T2) symptom severity (DASS-D, DASS-A), positive mental health (PMH), and global success ratings (GSR), a series of hierarchical regression analyses was calculated using age, gender, DASS-D (T1), DASS-A (T1), PMH (T1), and all CTQ subscales (T1) as predictors. Assuming a medium-sized effect (*f* ^2^ = 0.15), an alpha error level of 5%, 11 predictors, and a sample size of *N*= 549, the test power was 1−ß ≥ 0.90 and therefore sufficient according to Cohen (19). In all hierarchical regression models, there was no violation of the multicollinearity assumption, as all values of tolerance were >.25, and all variance inflation factor values were <5 (20).

## Results

In total, 57.6% (*n* = 316) participants suffered from some kind of childhood maltreatment. Emotional abuse was reported by 43.5% participants (none/mild = 41.2%; low/moderate = 29.2%; moderate/severe = 10.0%; severe/extreme = 19.5%). Physical abuse was reported by 20.8% participants (none/mild = 79.2%; low/moderate = 8.6%; moderate/severe = 5.5%; severe/extreme = 6.8%). Emotional neglect was reported by 34.2% participants (none/mild = 33.9; low/moderate = 31.9%; moderate/severe = 15%; severe/extreme = 19.2%), and physical neglect was reported by 36.9% participants (none/mild = 63.1%; low/moderate = 18.3%; moderate/severe = 13.3%; severe/extreme = 5.3%).

*Childhood maltreatment prevalence rates in regard to gender and diagnostic groups.* Female participants suffered from more experiences with emotional abuse, *t (546)* = 3.81, *p* <.001, *d* = 0.33; sexual abuse, *t*(543) = 2.31, *p* = .01, *d* = 0.20; and physical neglect, *t*(545) = 2.21, *p* = .01, *d* = 0.19, than male participants (see also **Table 1**).

**Table 1:** Descriptive statistics and prevalence rates of CT at T1.

	*n*	Emotional abuse		Physical abuse		Emotional neglect		Physical neglect		Sexual abuse*
		*M* (*SD*)	%	*M* (SD)	%	*M* (*SD*)	%	*M* (*SD*)	%	*M* (*SD*)
Total sample	549	10.43 (5.43)	43.5	6.65 (3.18)	20.8	12.37 (5.26)	34.2	7.35 (2.81)	36.9	4.83 (2.34)
Gender										
Male	219	9.35 (4.84)	34.9	6.63 (3.14)	21.2	11.97 (4.93)	29.0	7.03 (2.55)	29.8	4.55 (1.99)
Female	330	11.12 (5.68)	49.4	6.68 (3.24)	20.6	12.63 (5.47)	37.7	7.57 (2.95)	41.6	5.02 (2.54)
Diagnosis										
MDE, single	55	10.72 (5.36)	45.5	7.23 (3.32)	29.1	13.03 (5.08)	41.8	8.12 (3.26)	50.9	5.10 (3.05)
MDE, recurrent	120	11.15 (5.95)	46.7	7.05 (3.66)	25.2	13.04 (5.16)	41.7	7.70 (2.82)	47.5	4.92 (2.24)
Dysthymia	43	11.02 (5.54)	48.8	7.00 (3.28)	27.9	14.41 (6.09)	41.9	7.95 (2.58)	53.5	4.88 (2.04)
Panic disorder with/without agoraphobia	50	9.26 (5.12)	32.0	6.36 (2.50)	22.0	11.02 (5.22)	26.0	6.66 (2.39)	24.0	4.18 (0.62)
Social phobia	52	11.09 (5.82)	51.9	6.55 (3.00)	21.2	13.36 (5.30)	42.3	7.90 (3.48)	38.5	4.94 (2.75)
Adjustment disorder	60	9.50 (4.39)	38.3	5.71 (1.35)	8.3	11.51 (4.93)	33.3	6.96 (2.23)	31.7	4.50 (1.51)

MDE, major depression episode. *One item of the CTQ assessing sexual abuse was missing; therefore, no prevalence rates are reported on sexual abuse. Prevalence rates are reported using cut-off scores by Walker et al. (12).

On a descriptive level, emotional abuse and emotional neglect were most prevalent in participants suffering from social phobia, whereas physical abuse and physical neglect were most prevalent in participants suffering from some kind of unipolar depression. In a series of multiple logistic regression models (see **Table 2**), Social Phobia, Dysthymia, and Major Depression (single episode) were most strongly associated (OR ≥2) with emotional neglect, whereas Dysthymia, Major Depression (single/recurrent), and Social Phobia were most strongly associated (OR ≥2) with physical neglect. None of the different disorders showed a notable association with emotional or physical abuse.

**Table 2:** Multiple logistic regression analysis predicting CT.

	Emotional abuse			Physical abuse			Emotional neglect			Physical neglect		
	OR	(95% CI)	*p*	OR	(95% CI)	*p*	OR	(95% CI)	*p*	OR	(95% CI)	*p*
Gender	0.50	0.345–0.720	.001	1.02	0.661–1.587	.914	0.60	0.413–0.893	.011	0.52	0.359–0.776	.001
Age	1.00	0.987–1.010	.952	1.02	1.011–1.044	.001	1.02	1.006–1.035	.005	1.01	1.001–1.029	.032
MDE, single	1.12	0.609–2.105	.696	1.92	0.944–3.943	.072	2.14	1.118–4.105	.022	2.95	1.553–5.606	.001
MDE, recurrent	1.24	0.764–2.019	.383	1.35	0.751–2.450	.312	1.97	1.172–3.313	.011	2.45	1.472–4.110	.001
Dysthymia	1.57	0.787–3.163	.198	1.55	0.700–3.446	.279	2.20	1.068–4.538	.033	3.60	1.756–7.397	.001
Panic disorder with/without agoraphobia	0.62	0.319–1.234	.177	1.34	0.610–2.954	.465	1.03	0.497–2.155	.928	0.87	0.413–1.834	.714
Social phobia	1.71	0.906–3.258	.098	1.42	0.647–3.148	.378	2.69	1.375–5.271	.004	2.16	1.100–4.248	.025
Adjustment disorder	0.82	0.443–1.153	.544	0.37	0.136–1.034	.058	1.31	0.675–2.547	.424	1.26	0.635–2.461	.480

MDE, major depression episode; OR, odds ratios. Odds ratios represent differences to the mean effect in the current analysis.

*Childhood maltreatment and symptom severity*. The presence of any kind of childhood maltreatment, except for sexual abuse, was associated with more severe pretreatment depression and anxiety as well as lower levels of positive mental health (see **Table 3**).

**Table 3:** Associations between pretreatment symptom severity and experiences with childhood maltreatment.

	Emotional abuse	Physical abuse	Sexual abuse	Emotional neglect	Physical neglect
	*r*	*r*		*r*	*r*
DASS-D	.260**	.093*	0.069	.223**	.185**
DASS-A	.230**	.190**	0.055	.156**	.167**
PMH	**−**.254**	**−**.098*	−0.44	**−**.282**	**−**.198**

**p <.01; *p <.05.

*Childhood maltreatment and treatment outcome*. T-tests for dependent samples revealed that depression (T1: *M* = 19.60, *SD* = 11.14; T2: *M* = 13.26, *SD* = 10.85), *t*(543) = 14.36, p ≤.001, *d* = .62, and anxiety (T1: *M* = 11.64, *SD* = 7.92; T2: *M* = 8.23, *SD* = 7.36), *t*(546) = 10.58, p ≤.001, *d* = 36, decreased whereas positive mental health (T1: *M* = 10.30, *SD* = 5.55; T2: *M* = 13.47, *SD* = 6.22), *t*(536) = 15.21, p ≤.001, *d* = 66, increased from before to after treatment. **Tables 4**–**7** show the results of hierarchical linear regression analyses with posttreatment depression, anxiety, positive mental health, and global success as criterion variables. *Emotional abuse* was predictive of heightened posttreatment depression (see **Table 4**), posttreatment anxiety (see **Table 5**), and lowered posttreatment positive mental health (see **Table 6**)—after control of age, gender, pretreatment depression, anxiety, and positive mental health. *Emotional neglect* was predictive of lower patient posttreatment success rating (see **Table 7**). *Sexual abuse*, *physical abuse*, and *physical neglect* showed no predictive association in any of the investigated models. Of note, pretreatment positive mental health was shown to be predictive of therapy outcome in every investigated model. However, it has to be highlighted that adding the CTQ subscales in step 2 of each model only improved the model’s explained variance for posttreatment depression (see **Table 4**), but not for posttreatment anxiety, posttreatment positive mental health, and global success (see **Tables 5**–**7**).

**Table 4:** Prediction of posttreatment depression by pretreatment symptomatology, positive mental health, and traumatic childhood experiences (*n* = 528).

	Model 1			Model 2		
Pretreatment characteristics	B	T	p	B	T	p
Age	.041	1.16	.243	.050	1.37	.169
Gender	−.015	−0.44	.658	.013	036	.714
DASS-D	.346	6.55	.001	.331	6.29	.001
DASS-A	−.012	−0.31	.755	−.023	−0.56	.570
PMH	−.317	−6.36	.001	−.295	−5.88	.001
CTQ-EA	–	–	–	.147	2.70	.007
CTQ-PA	–	–	–	−.061	−1.37	.170
CTQ-SA	–	–	–	−.015	−0.39	.690
CTQ-EN	–	–	–	−.020	−0.35	.723
CTQ-PN				.051	0.96	.337
Model	Adj. R^2^ = .362 *F*(5, 522) = 62.04 *p* <.001			Adj. R^2^ = .373 *F*(10, 517) = 32.96 *p* <.001		
Change in R^2^	0.367*p* <.001			0.017*p* <.016		

CTQ-EA, Childhood Trauma Questionnaire—Emotional Abuse; CTQ-PA, Childhood Trauma Questionnaire—Physical Abuse; CTQ-SA, Childhood Trauma Questionnaire—Sexual Abuse; CTQ-EN, Childhood Trauma Questionnaire—Emotional Neglect; CTQ-PN, Childhood Trauma Questionnaire—Physical Neglect; DASS-D, Depression Anxiety Stress Scales—Depression Subscale; DASS-A, Depression Anxiety Stress Scales—Anxiety Subscale; PMH, Positive Mental Health Scale.

**Table 5:** Prediction of posttreatment anxiety by pretreatment symptomatology, positive mental health, and traumatic childhood experiences (*n* = 531).

	Model 1			Model 2		
Pretreatment characteristics	B	T	p	B	T	p
Age	.073	2.30	.022	.088	2.65	.008
Gender	−.060	−1.88	.060	−.043	−1.32	.187
DASS-D	−.120	−2.48	.013	−.128	−2.65	.008
DASS-A	.629	17.44	.001	.621	16.99	.001
PMH	−.243	−5.33	.001	−.242	−5.23	.001
CTQ-EA	–	–	–	.125	2.50	.013
CTQ-PA	–	–	–	−.008	−0.19	.848
CTQ-SA	–	–	–	.012	0.33	.736
CTQ-EN	–	–	–	−.074	−1.42	.156
CTQ-PN				−.009	−0.18	.853
Model	Adj. R^2^ = .468 *F*(5, 525) = 94.33 *p* <.001			Adj. R^2^ = .471 *F*(10, 520) = 48.13 *p* <.001		
Change in R^2^	0.468 *p* <.001			0.007 *p = .*189		

CTQ-EA, Childhood Trauma Questionnaire—Emotional Abuse; CTQ-PA, Childhood Trauma Questionnaire—Physical Abuse; CTQ-SA, Childhood Trauma Questionnaire—Sexual Abuse; CTQ-EN, Childhood Trauma Questionnaire—Emotional Neglect; CTQ-PN, Childhood Trauma Questionnaire—Physical Neglect; DASS-D, Depression Anxiety Stress Scales—Depression Subscale; DASS-A, Depression Anxiety Stress Scales—Anxiety Subscale; PMH, Positive Mental Health Scale.

**Table 6:** Prediction of posttreatment positive mental health by pretreatment symptomatology, positive mental health, and traumatic childhood experiences (*n* = 529).

Pretreatment characteristics	Model 1			Model 2		
	B	T	p	B	T	p
Age	−.088	−2.76	.006	−.091	−2.73	.006
Gender	.024	0.75	.448	.013	0.41	.682
DASS-D	−.083	−1.70	.088	−.077	−1.60	.110
DASS-A	−.013	−0.36	.716	−.006	−0.15	.879
PMH	.610	13.35	.001	.591	12.78	.001
CTQ-EA	–	–	–	−.099	−1.97	.048
CTQ-PA	–	–	–	.008	0.18	.854
CTQ-SA	–	–	–	.060	1.72	.085
CTQ-EN	–	–	–	−.029	−0.55	.579
CTQ-PN				.026	0.54	.588
Model	Adj. R^2^ = .466 *F*(5, 523) = 93.12 *p* <.001			Adj. R^2^ = .471 *F*(10, 518) = 48.05 *p* <.001		
Change in R^2^	0.471 *p* <.001			0.010 *p = .*070		

CTQ-EA, Childhood Trauma Questionnaire—Emotional Abuse; CTQ-PA, Childhood Trauma Questionnaire—Physical Abuse; CTQ-SA, Childhood Trauma Questionnaire—Sexual Abuse; CTQ-EN, Childhood Trauma Questionnaire—Emotional Neglect; CTQ-PN, Childhood Trauma Questionnaire—Physical Neglect; DASS-D, Depression Anxiety Stress Scales—Depression Subscale; DASS-A, Depression Anxiety Stress Scales—Anxiety Subscale; PMH, Positive Mental Health Scale.

**Table 7:** Prediction of Global Success by symptomatology, positive mental health, and traumatic childhood experiences (*n* = 530).

Pretreatment characteristics	Model 1			Model 2		
	B	T	p	B	T	p
Age	−.054	−1.32	.185	−.030	−0.69	.489
Gender	−.066	−1.61	.106	−.068	−1.60	.110
DASS-D	−.054	−0.86	.390	−.054	−0.86	.390
DASS-A	.058	1.24	.213	.052	1.09	.274
PMH	.312	5.31	.001	.284	4.77	.001
CTQ-EA	–	–	–	.072	1.11	.265
CTQ-PA	–	–	–	.043	0.82	.412
CTQ-SA	–	–	–	.004	0.09	.928
CTQ-EN	–	–	–	−.186	−2.77	.006
CTQ-PN				.011	0.18	.856
Model	Adj. R^2^ = .117*F*(5, 524) = 14.95*p* <.001			Adj. R^2^ = .126*F*(10, 519) = 8.60*p* <.001		
Change in R^2^	0.125 *p* <.001			0.017 *p = .*065		

CTQ-EA, Childhood Trauma Questionnaire—Emotional Abuse; CTQ-PA, Childhood Trauma Questionnaire—Physical Abuse; CTQ-SA, Childhood Trauma Questionnaire—Sexual Abuse; CTQ-EN, Childhood Trauma Questionnaire—Emotional Neglect; CTQ-PN, Childhood Trauma Questionnaire—Physical Neglect; DASS-D, Depression Anxiety Stress Scales—Depression Subscale; DASS-A, Depression Anxiety Stress Scales—Anxiety Subscale; GSR, Global Success Rating; PMH, Positive Mental Health Scale.

In a series of exploratory analysis, it could be shown that all result patterns remain the same when entering Posttraumatic Stress Disorder (PTSD) diagnosis (yes/no) as a further predictor variable.

## Discussion

In line with previous studies (5, 6), the present study found a high burden of childhood maltreatment, particularly emotional abuse and neglect (7), in outpatient psychotherapy patients. Some form of maltreatment was reported by 57.6% of the study sample. Out of the participants with childhood maltreatment, the majority scored in the mild childhood trauma index range (31.9% to 79.2%), yet 5.2% to 19.5% scored in the extreme range. Women were more affected than men (9), and in the context of social phobias and unipolar depression, particularly close links were found with childhood neglect (21). Furthermore, the study results complement previous studies showing an association between childhood maltreatment and symptom severity (5, 6) as well as reduced optimism (21). In this sense, the present study found that individuals who reported any type of childhood maltreatment at baseline, except for sexual abuse, suffered from higher levels of depression and anxiety severity as well as reduced positive mental health. The results underscore the corrosive effect of childhood maltreatment. The finding on sexual abuse may be related to the fact that such experiences were rather seldom in the present study sample.

In terms of treatment response, self-reported emotional abuse was predictive of increased posttreatment depression, anxiety, and reduced positive mental health. Emotional neglect was predictive of lower patient-reported global treatment success. The influence of childhood maltreatment on treatment outcome is small in size (9); however, all associations were shown when age, gender, pretreatment depression, anxiety, and positive mental health were controlled for. With regard to routine short-term psychotherapeutic care, it was thus shown—as in other studies (6, 10)—that childhood maltreatment is associated with a lower response to psychotherapy. The corresponding effect was shown in relation to a sample of patients suffering from a variety of diagnoses and receiving short-term cognitive behavioral therapy in a routine care context. Of note, the effect was only found for forms of emotional abuse or emotional neglect, whereas no corresponding effects were shown for sexual abuse or physical abuse/neglect. This finding is consistent with earlier study findings, showing that from all types of childhood maltreatment, emotional abuse and/or emotional neglect seemed to show the most profound effect on personality characteristics, negative cognition, stress system dysregulation, and poorer lifestyle (7). It has been speculated that “the explanations for the particularly strong link between emotional maltreatment and negative cognitions is that in the case of emotional abuse or emotional neglect, negative self-associations are explicitly handed to the child by the parent (e.g., “you are worthless”)” (7). However, it might be necessary to further differentiate between emotional neglect and emotional abuse (22): emotional neglect is characterized by *deprivation* in attachment, emotional validation, and interpersonal stimulation, whereas emotional abuse is characterized by *threat* to one’s personal integrity. It has been shown that deprivation is, for example, associated with diminished verbal fluency (23) as well as anhedonia (24), whereas threat is, for example, associated with avoidance (25). Against this background, it might be speculated that experiences with deprivation and threat affect the therapeutic process and therapy response in different ways. In line with this, emotional neglect and emotional abuse are predictive of different types of therapy outcome in the current study. However, no far-reaching conclusions should be drawn until this pattern of findings has been replicated. However, a closer examination of the underlying mechanisms by which different types of childhood maltreatment affect the treatment process is urgently needed. Interestingly—and as a secondary finding—positive mental health, as measured by the PMH scale (15), was found to be predictive of every single treatment outcome measure assessed in this study. This is consistent with previous studies in which PMH was found to be a predictor of treatment outcome after exposure therapy (26–28) and points to the considerable importance of resilience factors, such as positive mental health, in overcoming psychological distress.

From a clinical–therapeutic perspective, the results point to the need for therapists to always assess whether and to what extent childhood maltreatment is reported. In routine practice, the Childhood Trauma Screener (29)—a short form of the CTQ with five items—might be used to inform case conceptualization and instigate further assessment of childhood maltreatment in the early phase of therapy. Particular attention should be paid here to emotional abuse/neglect, i.e., forms of maltreatment that may be less obvious and therefore—unlike serious sexual or physical abuse—potentially less clearly represent the focus of complaints. Exposure to childhood maltreatment—especially emotional abuse/neglect—may alter basic cognitive assumptions about the self and others, which over time may become ingrained in an individual’s personality (7). This stronger internalization of negative self-assumptions may require adapted therapy methods (e.g., emotion-focused/experiential interventions, autobiography-oriented interventions, more repetition), which should be taken into account when planning treatment. In contrast to traumatization that manifests itself in Posttraumatic Stress Disorder, however, there are fewer clear treatment recommendations for the more subtle forms of maltreatment. More research is needed here. In this context, it would also be important to investigate whether patients with childhood maltreatment actually need adapted interventions or simply more therapy sessions.

There are several limitations relevant to the current study. First, it is important to be aware that the CTQ is a retrospective self-report measure of childhood maltreatment. This, as well as the fact that the use of different cut-off scores is associated with different maltreatment prevalence rates (30), underscores the need to regard reported maltreatment rates as approximate values. Second, the CTQ does not allow to assess the age at which maltreatment was experienced. It was therefore impossible to investigate whether maltreatment during specific time windows exerts a particularly negative influence (31). Third, only main effects of the different maltreatment types were considered in the present study. Since maltreatment types are highly interrelated and frequently occur together (32), it would be important in future studies to also investigate cumulative effects of different maltreatment types on treatment response. Fourth, only comparatively few different disorder diagnoses could be considered separately in the present study. It therefore remains unclear to what extent the findings found here can be generalized to patients suffering from other disorders. This is truer since assignment to a diagnostic group (yes/no) was based on the primary diagnosis in the current study and did not take comorbidities into account. In future studies on larger samples, it would be interesting to not only investigate a more diverse range of disorders but also investigate specific combinations of comorbid disorders. Fifth, due to a technical error—as already mentioned—no prevalence data on the frequency of sexual abuse could be provided. Nevertheless, sexual abuse experiences could still be taken into account in the majority of the analyses conducted. Finally, a specific form of operationalizing treatment outcome was used in the present regression analyses (33). In other studies, treatment outcome has been operationalized differently: some studies focus on response and/or remission rates (6, 9, 10), whereas other studies focus on standardized mean differences (34). All of these approaches may be justified, but it is important to have a close look at the operationalization of treatment outcomes when considering different studies together, especially as this may have an impact on the results (3, 9).

In sum, the current study highlights the importance of taking experiences with childhood maltreatment into account in psychotherapeutic routine care: Childhood maltreatment is very prevalent and associated with symptom severity as well as reduced treatment response in short-term psychotherapy. Emotional abuse and emotional neglect exert an especially pernicious influence; particular attention must therefore be paid to these respective childhood experiences, as they can easily go unnoticed in the early phases of psychotherapy.

## Data Availability

The raw data supporting the conclusions of this article will be made available by the authors, without undue reservation.
